# Publisher Correction: Detection of genetic variation and base modifications at base-pair resolution on both DNA and RNA

**DOI:** 10.1038/s42003-021-01894-9

**Published:** 2021-03-11

**Authors:** Zhen Wang, Jérôme Maluenda, Laurène Giraut, Thibault Vieille, Andréas Lefevre, David Salthouse, Gaël Radou, Rémi Moulinas, Sandra Astete, Pol D’Avezac, Geoff Smith, Charles André, Jean-François Allemand, David Bensimon, Vincent Croquette, Jimmy Ouellet, Gordon Hamilton

**Affiliations:** 1 Depixus SAS, Paris, France; 2 Laboratoire de physique de L’École normale supérieure de Paris, CNRS, ENS, Université PSL, Sorbonne Université, Université de Paris, Paris, France; 3 IBENS, Département de biologie, École normale supérieure, CNRS, INSERM, PSL Research University, Paris, France; 4 Department of Chemistry and Biochemistry, UCLA, Los Angeles, CA USA; 5 ESPCI Paris, PSL University, Paris, France

**Keywords:** RNA splicing, Single-molecule biophysics, RNA sequencing, Methylation analysis, Magnetic tweezers

Correction to: *Communications Biology* 10.1038/s42003-021-01648-7, published online 29 January 2021.

The original version of the Article contained an error in Fig. 5d, in which a white rectangle was introduced, obscuring part of the data in the row marked 588. The correct version of Fig. 5 is: which replaces the previous incorrect version. The error has been corrected in the HTML and PDF versions of the Article.

