# Supplementary material for: Detection of genetic variation and base modifications at base-pair resolution on both DNA and RNA
Source: Commun Biol. 2021 Jan 29;4:128. doi: 10.1038/s42003-021-01648-7 (PMC7846774; doi:10.1038/s42003-021-01648-7)
Supplement: Supplementary file 2 — Supplementary Information [file 42003_2021_1648_MOESM2_ESM.pdf]

Supplementary Information

Detection of genetic variation and base modifications at base-pair resolution on both DNA and RNA

Zhen Wang[1], Jérôme Maluenda[1], Laurène Giraut[1], Thibault Vieille[1], Andréas Lefevre[1], David Salthouse[1], Gaël Radou[1], Rémi Moulinas[1], Sandra Astete[1], Pol D'Avezac[1], Geoff Smith[1], Charles André[1], Jean-François Allemand[2,3], David Bensimon[2,3,4], Vincent Croquette[2,3,5], Jimmy Ouellet[1,6], Gordon Hamilton[1,6*]

[1] Depixus SAS, 3/5 Impasse Reille, 75014, Paris, France

[2] Laboratoire de Physique de l'École Normale Supérieure, ENS, Université PSL, CNRS, Sorbonne Université, Université Paris-Diderot, Sorbonne Paris Cité, Paris, France.

[3] IBENS, Département de biologie, École normale supérieure, CNRS, INSERM, PSL Research University, 75005 Paris, France.

[4] Department of Chemistry and Biochemistry, UCLA, 607 Charles E Young Drive East, Los Angeles, 90095, USA.

[5] ESPCI Paris, PSL University, 10 rue Vauquelin, 75005 Paris, France.

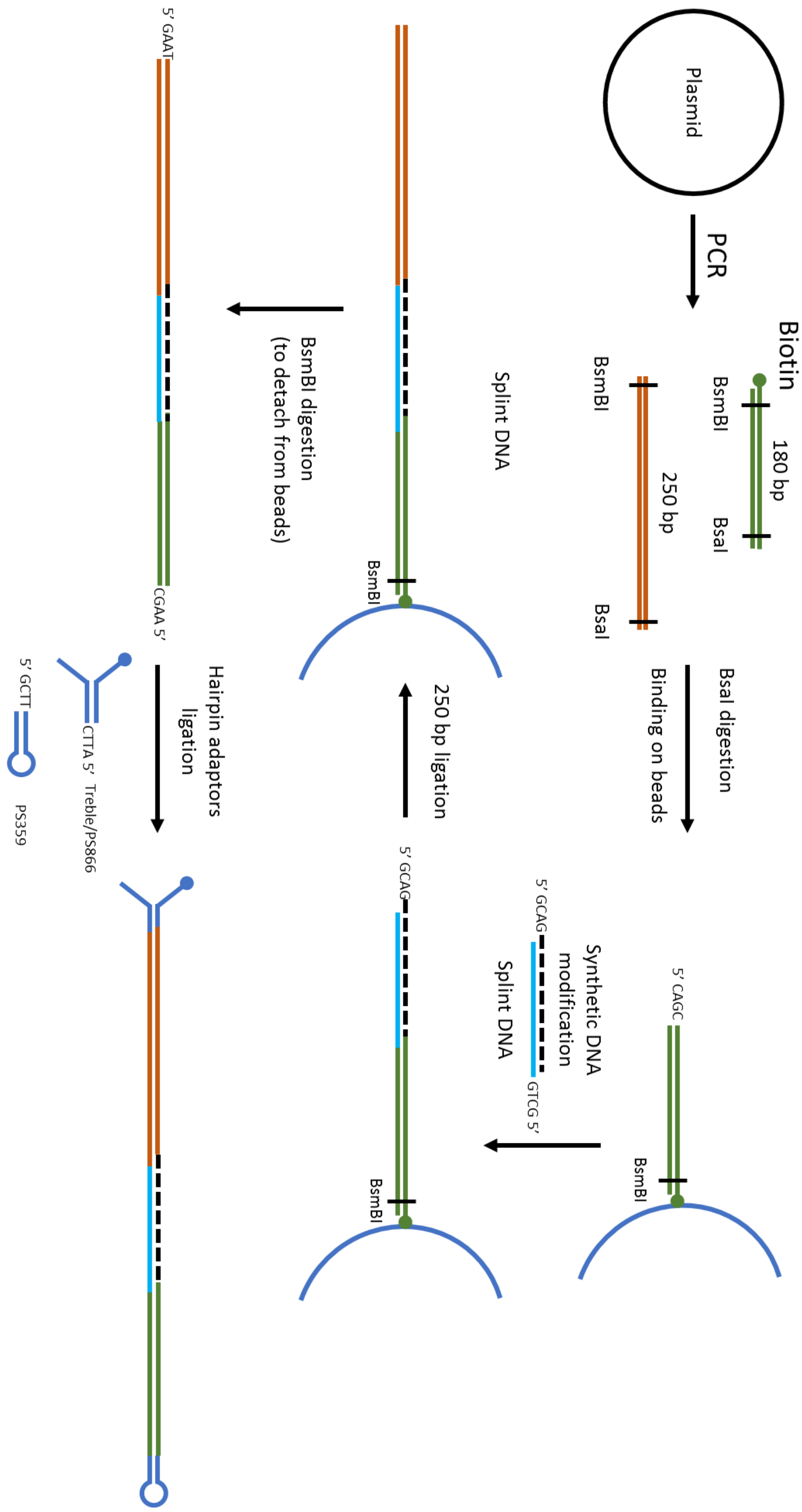

Supplementary Figure 1. Strategy for construction synthetic RNA and DNA hairpin containing epigenetic modifications from chemically synthesized oligonucleotides. Graphical representation of the strategy for assembling synthetic hairpins. Detailed protocol in the materials and methods section.

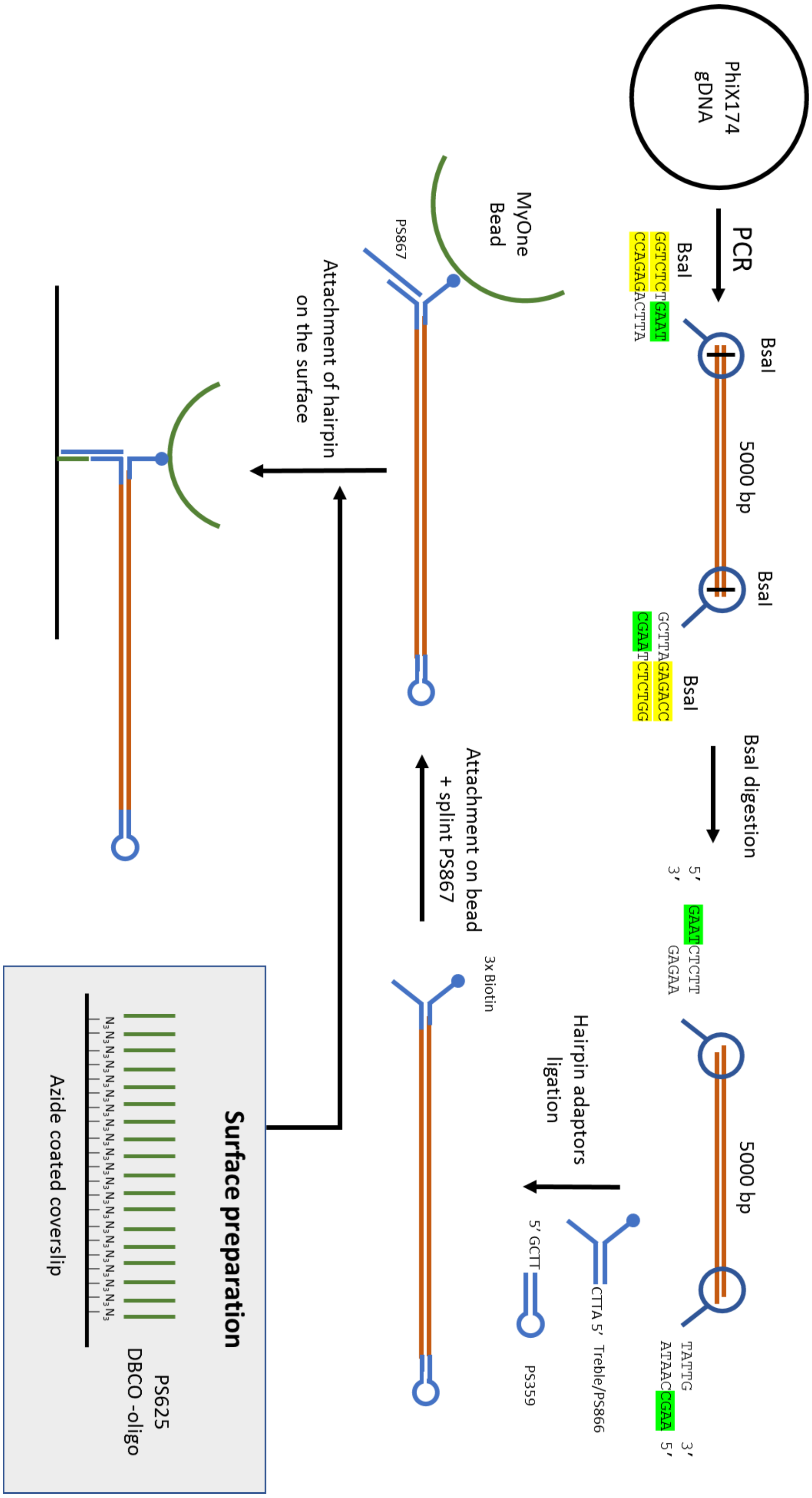

Supplementary Figure 2. Strategy for creating synthetic hairpins from PCR, followed by attachment on the surface. Details on the construction of this hairpin can be found in the materials and methods section.

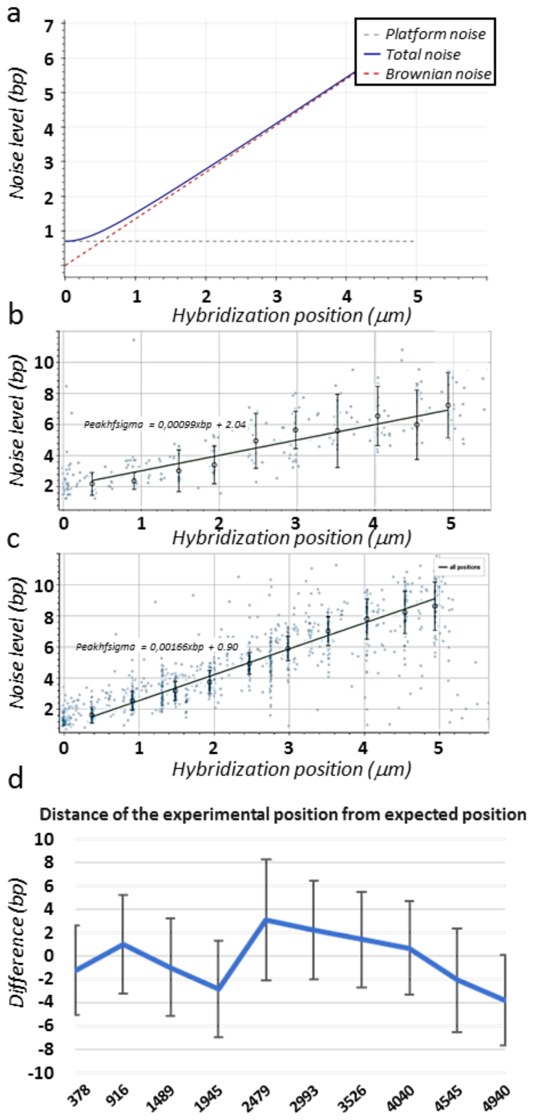

Supplementary Figure 3. Estimation of the platform noise. (a) Modeling of the measured total noise (dark blue line), which is composed of the platform noise (which is constant independently of the molecule length, black dotted line) plus the Brownian noise, which is dependent on the length of the molecule (red dotted line). A 5 kb hairpin was constructed to determine the platform noise by testing ten ten-base oligonucleotides along the length of the molecules. For each position, the noise was recorded at a frequency of 30 images per second and plotted against the position of blockage (each bleu point corresponds to a mapped blocking position). After fitting a linear regression, it is possible to determine the instrument noise (when the molecule length is 0), which is 2.04 bases for the original MT **(b)** and 0.90 bases for the new SDI platform **(c)**. **(d)** The difference in base pairs between the experimental and expected position for all the oligonucleotides along the 5 kb hairpin is plotted. For positions up to 1.5 kb from the Y-shape, the precision is less than 1 base and reached four bases at the 4940 bp position of the hairpin.

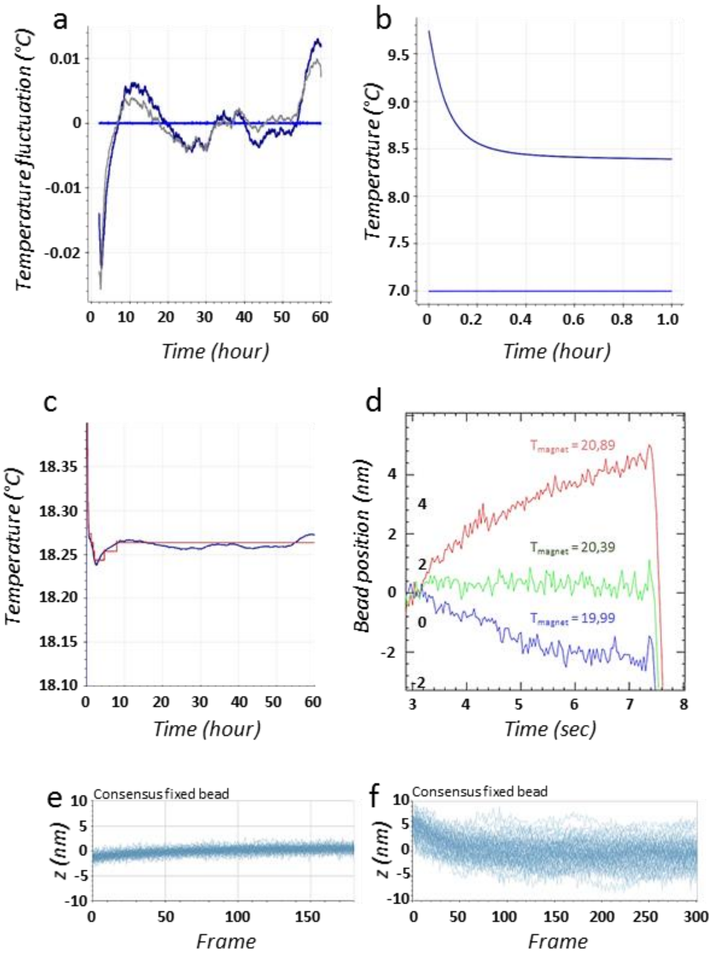

Supplementary Figure 4. Thermal control of the SDI instrument. (**a**) Long term recording (more than 60 hours) of the temperature of the SDI instrument at three different positions within the instrument, rescale to 0 degree – Dark blue corresponds to the variation in temperature of the box containing all the Peltier elements. The light blue correspond to the variation of the temperature of the sample and the grey line represents the variation in temperature inside the box (ambient temperature). There was a slow variation observed over few hours, with a maximal amplitude of 0,01°C. (**b**) The SDI instrument was able to reach a temperature of the sample of 8,5°C (dark blue) and remained stable at that temperature over hours. This represented a difference of 14°C compared to room temperature. (**c**) The magnets were also thermalized (dark blue line) and their temperature adapted to the sample temperature (red line, the temperature was decreased by steps of 0,01 °C within the first 10 hours). Although their temperature slightly varied, they stayed within 0,01°C from the temperature of the sample. (**d**) The temperature of the magnet can be adapted to limit the thermal drift observed during an experiment. For a temperature of the sample at 21°C, there was no drift in the position of the bead when the temperature of the magnets was set to 20,39°C. However, just an increase of 0,5°C in the temperature of the magnets introduced a drift of four nanometers and -2 nm when the temperature is decreased by 0,4°C. A consensus fixed bead was created on the SDI (**e**) and on the previous magnetic tweezer instrument (**f**) and both the noise level and the distribution of the signal were tighter with the new SDI instrument.

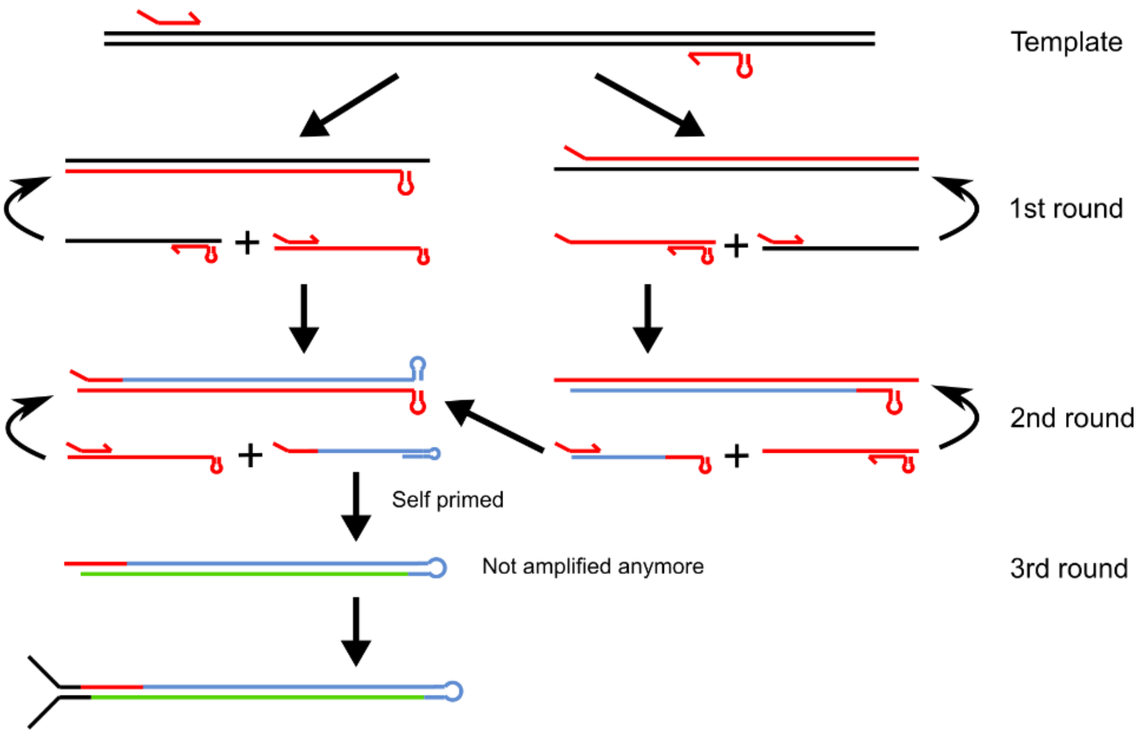

Supplementary Figure 5. Principle of the loop PCR strategy. The loop PCR strategy uses a normal forward primer, but the reverse primer is designed so that it encompasses a loop. At the first cycle of the PCR, the reverse looped primer creates a DNA molecule which incorporates the loop at its 5' end, which cannot be extended. In the second cycle, when the forward primer is extended, it will copy the loop generated in the previous cycle. In the third cycle, the replicated loop, which is now at the 3’ end of the transcript, will fold back on itself and self-prime. Since this newly synthesized molecule is in a hairpin form, it cannot be further amplified. The remaining single-stranded cDNAs with loops at their 5’ ends will act as templates to each form another 3' loop molecule, which leads to the desired double-stranded hairpin product.

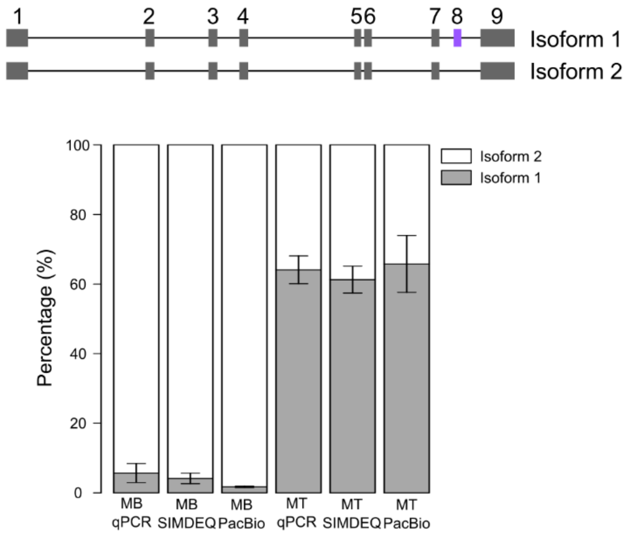

Supplementary Figure 6. Quantification of CAPZB in different cellular state using different techniques. Both isoforms of CAPZB were quantified in both myoblast and myotube for the inclusion or exclusion of exon 8. Similar results were obtained independently of the technique used for quantification.

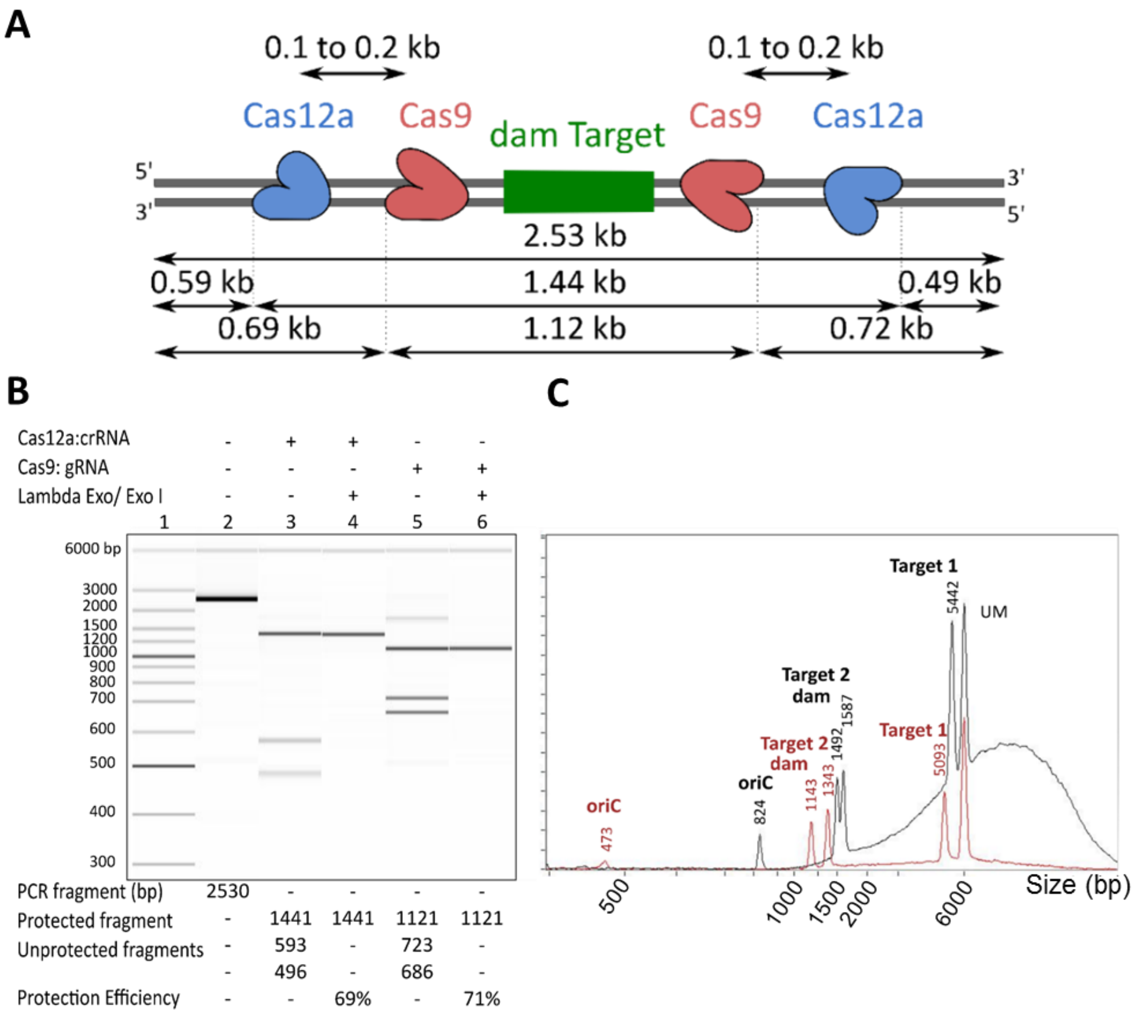

Supplementary Figure 7. Cas meganucleases can shield the end of DNA fragments from digestion with exonucleases. (A) Schematic representation of the experimental design of the protection assay. PCR fragment was generated such that the left- and right-side fragments resulting from digestion with Cas protein are of different sizes, allowing their simultaneous detection by capillary electrophoresis (schema not to scale). **(B)** The PCR product was incubated with either the Cas12a:crRNA complex (lanes 3-4) or the cas9:gRNA complex (lanes 5-6). After one hour of incubation, the reaction was supplemented with lambda Exo/ExoI nucleases (lanes 4 and 6) to digest all the DNA located outside the two Cas complexes. The expected fragments sizes and protection efficacy for each complex are listed below the Figure. **(C)** The protection of four targets from *E. coli* was achieved by incubating genomic DNA with all eight Cas12a:crRNA complexes corresponding to the four targets and after one hour, the reaction was supplemented with a mix of exonucleases (black outline). After this first protection step, 1/10th of the protected material was incubated with the eight dCas9:gRNA complexes and after one hour of incubation, exonucleases were added to the reaction tube (red outline). The peak corresponding to each target is marked as well as their estimated sizes. After the first protection step, a large quantity of undigested gDNA is present, indicated by the dome. This is eliminated with the second protection step. RFU: relative fluorescence unit. UM: Upper marker.

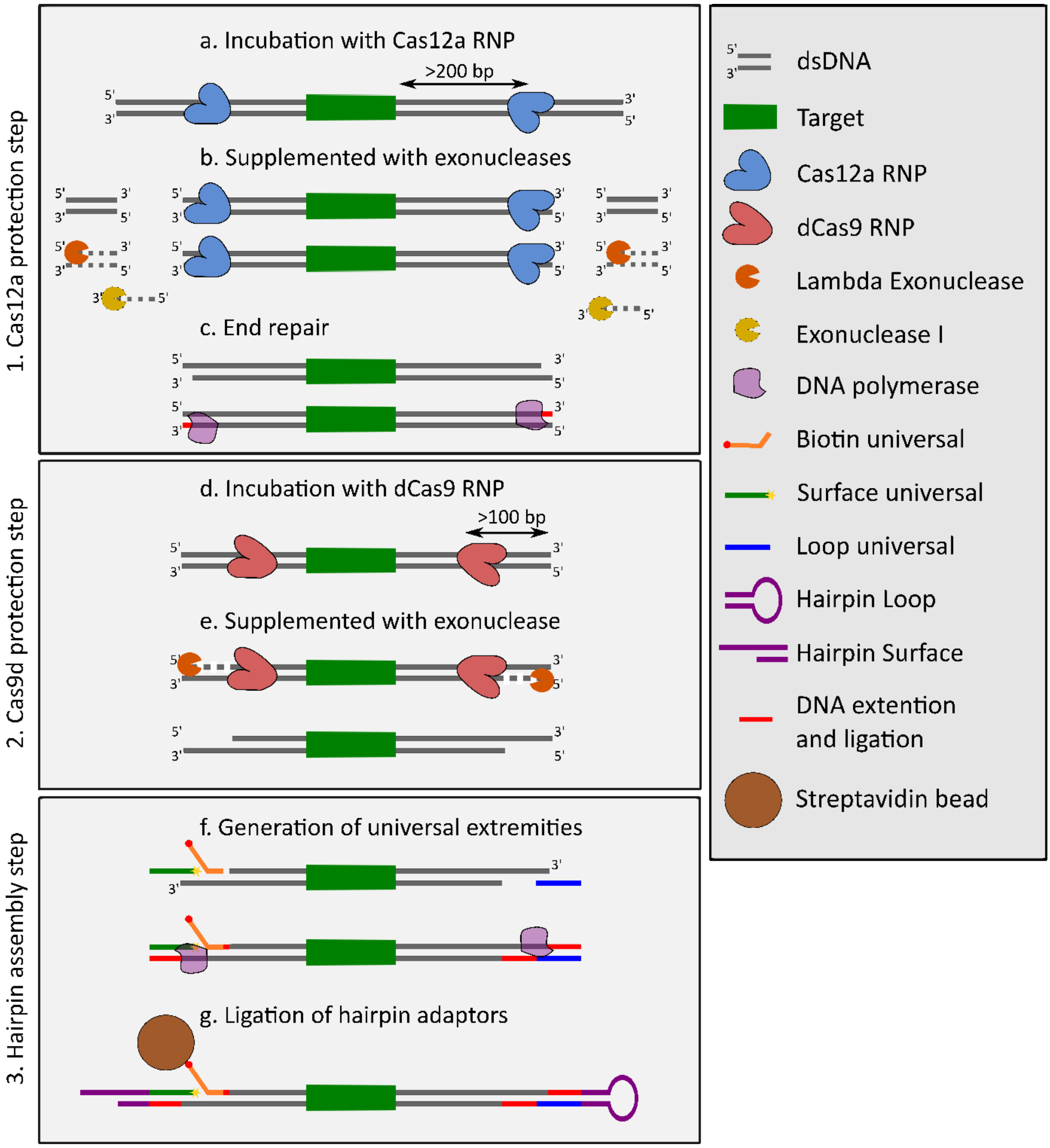

Supplementary Figure 8. A detailed schematic representation of the amplification-free enrichment protocol (1) Cas12a protection step. (a) dsDNA is incubated with two preloaded Cas12a and crRNA per targets for 1 hour at 37°C. Those two Cas12a/crRNA (Cas12a RNP) are design to flank the region of interest at least 200 bp from the target with the PAM sequence (TTTV) facing the target. **(b)** After one hour of incubation, the reaction is supplemented with a cocktail of exonuclease (lambda exonuclease and exonuclease I) and incubated for 1 hour at 37°C. These exonucleases will degrade all the DNA which is not protected by a Cas12a on each side. The reaction is then stop using proteases and purified using beads purification method. **(c)** The overhang created by the cleavage with Cas12a is repaired by T4 DNA polymerase. The reaction is then purified using bead purification method. **(2) dCas9 protection step. (d)** The resulting protected dsDNA is incubated with two preloaded dCas9 and tracr:crRNA per targets for 1 hour at 37°C. Those two dCas9/tracr:crRNA (dCas9 RNP) are design such that they are located at least 100 bp for the Cas12a site, with the PAM sequence (NGG) facing the target. **(e)** After incubation, the reaction is supplemented with lambda

exonuclease and incubated for 1 hour at 37°C. This will generate a 3' ssDNA extremities through the degradation of the 5' strand on each side of the DNA molecule. The dCas9 RNP serve as a roadblock of the 5' strand degradation. The reaction is purified by beads purification method. **(3) Hairpin assembly step. (f)** Universal extremities are generated by the hybridization of target specific oligonucleotides. *On one side of the molecule (right on the schematic)*: one oligonucleotide, called loop universal (blue), will bind to the 3' end. The polymerase (Bst Full Length) combined with a DNA Ligase (Taq DNA Ligase) will fill the gap between the oligonucleotide and the 5' DNA target end, while also filling the 5' tail of the oligonucleotide (which contain a non-palindromic restriction site). *On the other side of the molecule (left on the schematic)*: two oligonucleotides are bound on the ssDNA overhang. The first one (green), called surface universal, encompasses a 3' phosphate group (yellow star), which prevent to fill the gap between the oligonucleotide and the DNA target will bind at the 3' end of the DNA. However, the 5' tail of the oligonucleotide will be filled by using the 3', end of the fragment (which contain a non-palindromic restriction site with a different overhang than the other side). The second oligonucleotide (orange), called biotin universal, contains a 5' biotin group (red dot) and is complementary to the ssDNA (few bases after the surface universal). The gap between the biotin oligonucleotide and the recessed 5' end of the DNA target is filled by the polymerase at the same time as the other end (loop side). The reaction is purified using beads purification method. **(g)** The two universal hairpin adaptors, hairpin loop and hairpin surface (dark purple), are added to the previously constructed molecule by first digestion of the universal and loop adaptor with the non-palindromic restriction enzyme (BsaI). After digestion, the reaction is directly bound on streptavidin beads. After washing, the ligation of those two adaptors with a DNA ligase (T4 DNA ligase) is performed directly on beads. Beads are washed and ready to be loaded within a flow cell for analysis. The sequence of all the Cas12a and dCas9 RNA guides as well as the oligonucleotides used for the assembly of the *E. coli* and human hairpin is listed in supplemental table 2, 3 and 4.

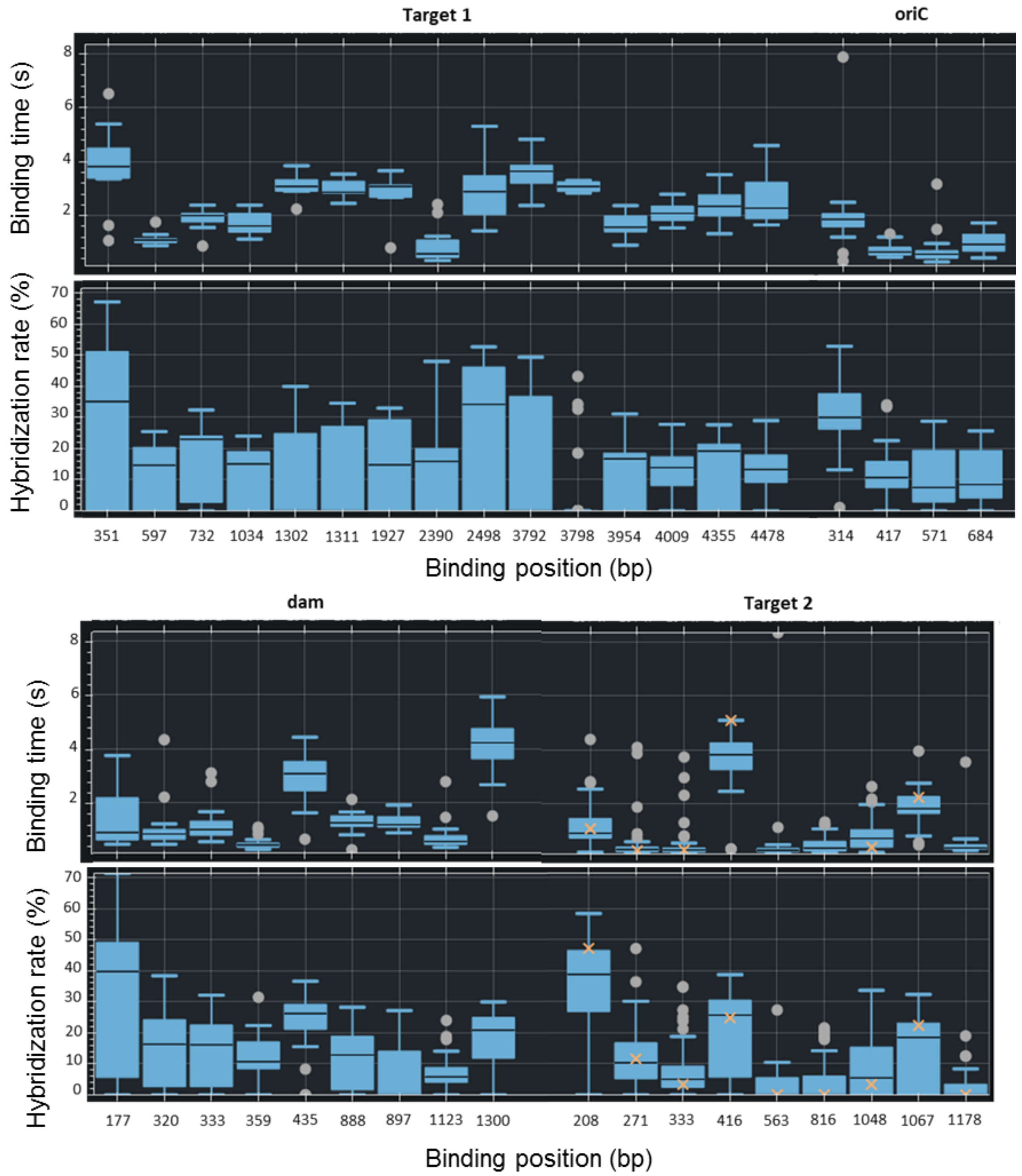

Supplementary Figure 9. Characterisation of CAAG binding on the four *E. coli* targets. For each position on each of the four targets, the binding time and the hybridization rate is indicated by a box plot.

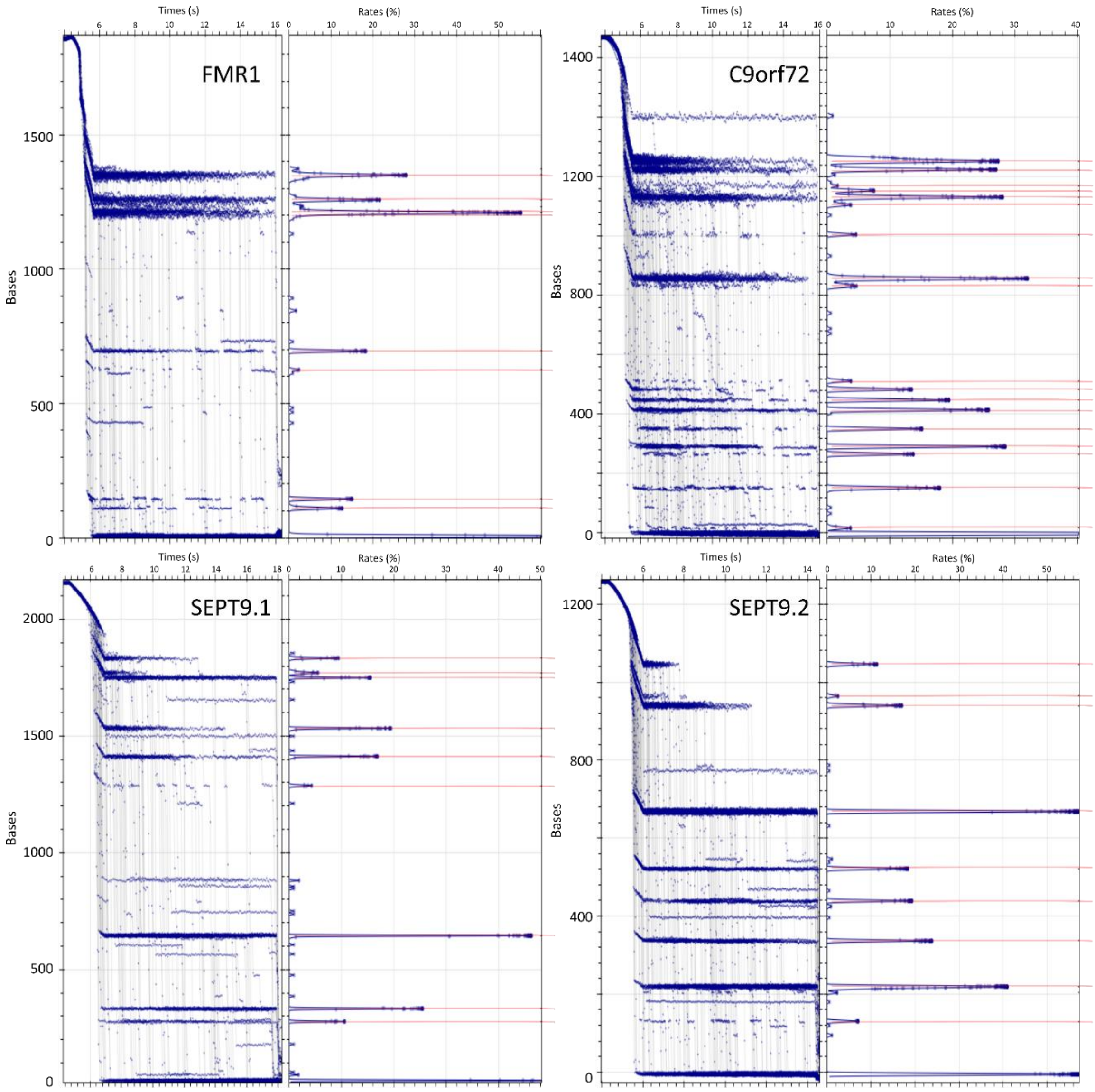

Supplementary Figure 10. Signature produced by the oligonucleotide CAAG on the enriched targets for human gDNA. Expected and experimental positions of the CAAG oligonucleotide on the FMR1, C9orf72, SEPT9.1 and SEPT9.2 hairpin produced from the enriched fragments. Each red line represents one expected blockage. These specific patterns allowed us to identify and classify the functional hairpins as one of the four targets.

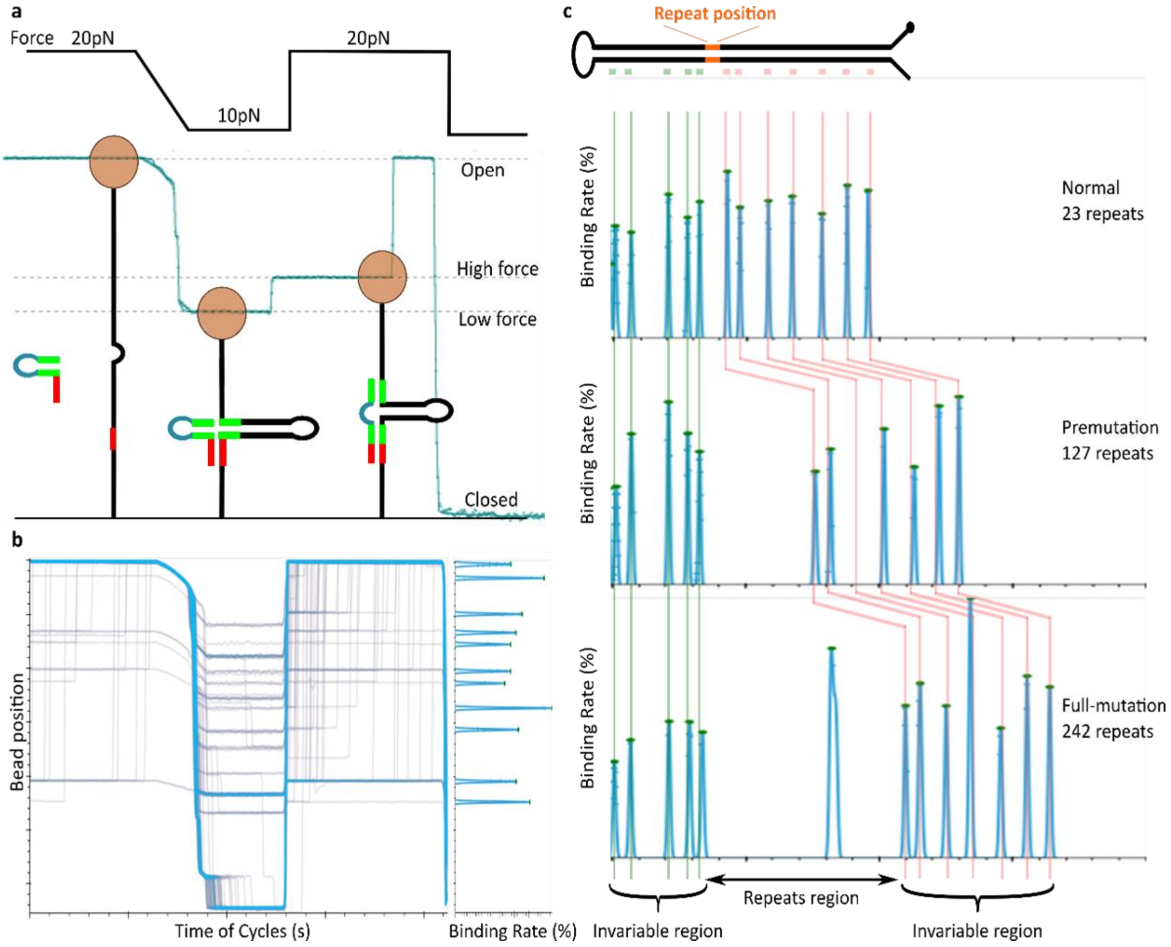

Supplementary Figure 11. Principle of the repeat analysis of FMR1 using an opening assay (A) Oligonucleotides designed to form a three-way junction on either side of the repeat region is injected into the flow cell (only one is shown) and the functional molecules are cycled between high and low forces. The single stranded part of the oligonucleotides binds to the molecules during the phase of high force (20 pN) and cause a transient blockage when the force is reduced (red part of the oligonucleotide on the complementary sequence in red on the hairpin). When the force is increased to 20 pN, a three-way junction is formed and transiently prevents opening of the molecules (the green sequence on the oligonucleotide is the same sequence as the hairpin, in green, causing strand invasion). Measurement at high force bypasses any secondary structures (for example, intra-molecule hairpins) that could affect the measurement of the blocking position during the closing phase. **(B)** Typical opening and closing cycles for this assay (left part) and the corresponding histogram of the detected blocking positions (right part). **(C)** Schematic representation of the invariable positions of the reference oligonucleotides that bind on either side of the repeat region of the FMR1 hairpin. Green lines represent the theoretical binding position of the five oligonucleotides located upstream of the repeats and the red lines are the positions located downstream of the repeats. The extracted experimental peaks were fitted to these reference positions (X-axis) and used to calibrate the measurement of the repeat length. The graph represents three different molecules with either 23, 127 or 242 repeats. (The additional peak within the repeat region of the molecule with 242 repeats is due to a non-specific blockage of the molecule within the repeats.)

Supplementary Table 1. CpG and Non-CpG sites on the FMR1 locus. List of all the positions and the rate of methylation across the molecule of the DNA sample NA06896.

				Methylated Molecules					
				WT n=11		Pre-Mutated n=9		Full-Mutated n=3	
Site Number	Position within HP	Sequence	Chr Position	Nb	Ratio	Nb	Ratio	Nb	Ratio
1	276	CG	147911340	1	0.09	0	0.00	0	0.00
2	300	CG	147911364	3	0.27	3	0.33	0	0.00
3	306	CG	147911370	1	0.09	3	0.33	0	0.00
4	321	CT	147911385	0	0.00	1	0.11	0	0.00
5	326	CG	147911390	2	0.18	1	0.11	0	0.00
6	346	CA	147911410	0	0.00	1	0.11	0	0.00
7	388	CT	147911452	0	0.00	2	0.22	0	0.00
8	397	CA	147911461	0	0.00	2	0.22	0	0.00
9	409	CG	147911473	3	0.27	2	0.22	1	0.33
10	428	CG	147911492	5	0.45	2	0.22	0	0.00
11	440	CT	147911504	0	0.00	1	0.11	0	0.00
12	443	CG	147911507	3	0.27	2	0.22	0	0.00
13	448	CT	147911512	0	0.00	1	0.11	0	0.00
14	456	CG	147911520	4	0.36	3	0.33	0	0.00
15	510	CG	147911574	4	0.36	4	0.44	0	0.00
16	512	CG	147911576	4	0.36	4	0.44	0	0.00
17	529	CG	147911593	3	0.27	2	0.22	1	0.33
18	543	CG	147911607	3	0.27	2	0.22	0	0.00
19	563	CG	147911627	6	0.55	2	0.22	0	0.00
20	593	CG	147911657	6	0.55	3	0.33	0	0.00
21	605	CG	147911669	5	0.45	2	0.22	0	0.00
22	607	CG	147911671	5	0.45	2	0.22	0	0.00
23	617	CG	147911681	4	0.36	3	0.33	0	0.00
24	625	CA	147911689	1	0.09	1	0.11	0	0.00
25	628	CG	147911692	5	0.45	5	0.56	0	0.00
26	639	CT	147911703	0	0.00	1	0.11	0	0.00
27	644	CG	147911708	5	0.45	4	0.44	0	0.00
28	660	CG	147911724	4	0.36	3	0.33	0	0.00
29	662	CG	147911726	3	0.27	3	0.33	0	0.00
30	666	CG	147911730	4	0.36	2	0.22	0	0.00
31	670	CG	147911734	2	0.18	3	0.33	0	0.00
32	675	CG	147911739	4	0.36	3	0.33	0	0.00
33	677	CG	147911741	4	0.36	3	0.33	0	0.00
34	679	CG	147911743	4	0.36	3	0.33	0	0.00
35	690	CG	147911754	5	0.45	2	0.22	0	0.00
36	695	CG	147911759	2	0.18	2	0.22	0	0.00
37	703	CG	147911767	4	0.36	2	0.22	0	0.00
38	707	CG	147911771	3	0.27	3	0.33	0	0.00
39	714	CA	147911778	1	0.09	2	0.22	0	0.00
40	720	CG	147911784	2	0.18	2	0.22	0	0.00
41	722	CG	147911786	1	0.09	2	0.22	0	0.00
42	728	CG	147911792	2	0.18	2	0.22	0	0.00
43	730	CG	147911794	2	0.18	0	0.00	0	0.00
44	732	CG	147911796	2	0.18	1	0.11	0	0.00
45	746	CA	147911810	0	0.00	3	0.33	0	0.00
46	750	CT	147911814	5	0.45	1	0.11	0	0.00
47	760	CG	147911824	5	0.45	4	0.44	0	0.00
48	766	CG	147911830	5	0.45	5	0.56	0	0.00
49	780	CG	147911844	3	0.27	3	0.33	0	0.00
50	782	CG	147911846	3	0.27	3	0.33	0	0.00
51	798	CG	147911862	3	0.27	4	0.44	0	0.00
52	807	CG	147911871	3	0.27	2	0.22	2	0.67
53	812	CG	147911876	4	0.36	2	0.22	2	0.67
54	815	CA	147911879	1	0.09	1	0.11	0	0.00
55	820	CA	147911884	3	0.27	2	0.22	0	0.00
56	829	CA	147911893	4	0.36	3	0.33	0	0.00
57	833	CG	147911897	3	0.27	2	0.22	0	0.00
58	838	CG	147911902	4	0.36	3	0.33	0	0.00
59	843	CG	147911907	3	0.27	4	0.44	0	0.00
60	851	CG	147911915	4	0.36	4	0.44	0	0.00
61	856	CA	147911920	2	0.18	4	0.44	0	0.00
62	865	CG	147911929	3	0.27	5	0.56	0	0.00
63	875	CG	147911939	6	0.55	6	0.67	0	0.00
64	880	CG	147911944	5	0.45	6	0.67	0	0.00
65	892	CG	147911956	4	0.36	5	0.56	0	0.00
66	899	CT	147911963	0	0.00	4	0.44	0	0.00
67	903	CG	147911967	4	0.36	5	0.56	0	0.00
68	913	CG	147911977	5	0.45	7	0.78	0	0.00
69	917	CG	147911981	4	0.36	4	0.44	0	0.00
70	920	CG	147911984	3	0.27	2	0.22	0	0.00
71	925	CG	147911989	4	0.36	5	0.56	0	0.00
72	928	CG	147911992	4	0.36	5	0.56	0	0.00
73	931	CG	147911995	5	0.45	3	0.33	0	0.00
74	935	CG	147911999	1	0.09	5	0.56	0	0.00
75	937	CG	147912001	0	0.00	2	0.22	0	0.00
76	941	CG	147912005	3	0.27	4	0.44	0	0.00
77	944	CG	147912008	3	0.27	3	0.33	0	0.00
78	947	CG	147912011	3	0.27	3	0.33	0	0.00
79	953	CG	147912017	4	0.36	5	0.56	0	0.00
80	957	CT	147912021	2	0.18	1	0.11	0	0.00
81	959	CG	147912023	4	0.36	3	0.33	0	0.00
82	962	CG	147912026	3	0.27	5	0.56	0	0.00
83	963	CT	147912027	3	0.27	2	0.22	0	0.00
84	968	CA	147912032	3	0.27	3	0.33	0	0.00
85	975	CG	147912039	4	0.36	3	0.33	0	0.00
86	979	CG	147912043	3	0.27	5	0.56	0	0.00
87	985	CG	147912049	4	0.36	5	0.56	0	0.00
