# Supplementary material for: Detection of genetic variation and base modifications at base-pair resolution on both DNA and RNA
Source: Commun Biol. 2021 Jan 29;4:128. doi: 10.1038/s42003-021-01648-7 (PMC7846774; doi:10.1038/s42003-021-01648-7)
Supplement: Supplementary file 3 — Description of Additional Supplementary Files [file 42003_2021_1648_MOESM3_ESM.pdf]

Description of Additional Supplementary Files

File name: Supplementary Data 1
Description: Experimental reagents. This file contains the sequence of all oligonucleotides used in this study as well as all Cas9 and Cas12 guides used for the enrichment of both E. coli and human genomic regions. This data is provided in the form of an Excel spreadsheet containing three tabs, one for each of oligonucleotides, Cas9 guides and Cas12 guides.

File name: Supplementary Data 2
Description: Excel file containing the source data underlying plots shown in the figures.
