# Supplementary material for: Detection of genetic variation and base modifications at base-pair resolution on both DNA and RNA
Source: Commun Biol. 2021 Jan 29;4:128. doi: 10.1038/s42003-021-01648-7 (PMC7846774; doi:10.1038/s42003-021-01648-7)
Supplement: Supplementary file 6 — Reporting Summary [file 42003_2021_1648_MOESM6_ESM.pdf]

nature research

Corresponding author(s): Gordon Hamilton

Last updated by author(s): Nov 17, 2020

Reporting Summary

Nature Research wishes to improve the reproducibility of the work that we publish. This form provides structure for consistency and transparency in reporting. For further information on Nature Research policies, see our Editorial Policies and the Editorial Policy Checklist.

Statistics

For all statistical analyses, confirm that the following items are present in the figure legend, table legend, main text, or Methods section.

n/a	Confirmed	
☐	☒	The exact sample size (n) for each experimental group/condition, given as a discrete number and unit of measurement
☐	☒	A statement on whether measurements were taken from distinct samples or whether the same sample was measured repeatedly
☐	☒	The statistical test(s) used AND whether they are one- or two-sided<br>*Only common tests should be described solely by name; describe more complex techniques in the Methods section.*
☒	☐	A description of all covariates tested
☒	☐	A description of any assumptions or corrections, such as tests of normality and adjustment for multiple comparisons
☐	☒	A full description of the statistical parameters including central tendency (e.g. means) or other basic estimates (e.g. regression coefficient) AND variation (e.g. standard deviation) or associated estimates of uncertainty (e.g. confidence intervals)
☒	☐	For null hypothesis testing, the test statistic (e.g. F, t, r) with confidence intervals, effect sizes, degrees of freedom and P value noted<br>*Give P values as exact values whenever suitable.*
☒	☐	For Bayesian analysis, information on the choice of priors and Markov chain Monte Carlo settings
☒	☐	For hierarchical and complex designs, identification of the appropriate level for tests and full reporting of outcomes
☒	☐	Estimates of effect sizes (e.g. Cohen's d, Pearson's r), indicating how they were calculated

Our web collection on statistics for biologists contains articles on many of the points above.

Software and code

Policy information about availability of computer code

Data collection	All raw data was collected using in-house software (known as Xvin) developed by authors VC and TV. This software is available on request.
Data analysis	Raw data were then processed using in-house signal-processing software and then extracted as experimental 'tracks' for further analysis. All the code used in this study is available on GitHub.

For manuscripts utilizing custom algorithms or software that are central to the research but not yet described in published literature, software must be made available to editors and reviewers. We strongly encourage code deposition in a community repository (e.g. GitHub). See the Nature Research guidelines for submitting code & software for further information.

Data

Policy information about availability of data

All manuscripts must include a data availability statement. This statement should provide the following information, where applicable:

- Accession codes, unique identifiers, or web links for publicly available datasets
- A list of figures that have associated raw data
- A description of any restrictions on data availability

All the requests for sequence, material or additional information should be addressed to Gordon Hamilton at gh@depixus.com

Field-specific reporting

Life sciences study design

All studies must disclose on these points even when the disclosure is negative.

Sample size	Sample sizes were not pre-determined before experiments. Typically data were generated and accumulated from molecules tracked across multiple camera fields of view. For a given sample, some variability was always seen in the numbers of analyzable molecules per field of view (FoV). And across samples and sample preparation approaches taken, there were also differences in the density of binding of molecules to the flow cell floor. We therefore did not analyze data from the same number of FoVs for all experiments.
Data exclusions	Due to the inherent variability of the pN force experienced by the beads (between 10-15% difference between beads), variability was also seen in the blockages caused by the binders. We therefore excluded data coming from molecules where we did not see all the blocking positions for the reference oligonucleotides that were used. Typically this meant removal of data from 30-40% of the tracked molecules.
Replication	The enrichment on E. coli was performed three times, each independently with different preparations of gDNA. For human enrichment, the enrichment using HEK gDNA was performed with multiple technical replicates, and for the Coriell gDNA sample (NA06896) with two technical replicates. For the analysis of epigenetic modifications, hairpins were constructed once and multiple FoVs were recorded with the antibodies. For the analysis of splicing variants, the cDNA library was prepared from three independent isolations of mRNA and three technical replicates were performed per cDNA library.
Randomization	N/A
Blinding	N/A

Reporting for specific materials, systems and methods

We require information from authors about some types of materials, experimental systems and methods used in many studies. Here, indicate whether each material, system or method listed is relevant to your study. If you are not sure if a list item applies to your research, read the appropriate section before selecting a response.

Materials & experimental systems

n/a	Involved in the study	
☐	☒	Antibodies
☐	☒	Eukaryotic cell lines
☒	☐	Palaeontology and archaeology
☒	☐	Animals and other organisms
☒	☐	Human research participants
☒	☐	Clinical data
☒	☐	Dual use research of concern

Methods

n/a	Involved in the study	
☒	☐	ChIP-seq
☒	☐	Flow cytometry
☒	☐	MRI-based neuroimaging

Antibodies

Antibodies used	For m5C, the ICC/IF (C15200003) mouse monoclonal antibody from Diagenode was used at 1:250. For m6A, we used the rabbit monoclonal m6A antibodies were from Cell Signaling Technology (clone D9D9W, at 1:500 dilution), the RevMAb Biosciences (clone RM362, at 1:300 dilution) and the mouse monoclonal recombinant AbFlex m6A antibody (rAb) from Active Motif (at 1:300 dilution). For 8-oxoG detection, we used the clone 15A3 from R&D Systems Europe Limited (4354-MC-050) at a dilution of 1:500. For hm5C, we used the clone RM236 from Invitrogen™ (15815913) at a dilution of 1:500. For ca5C detection, we used the clone RM24 1-A3 from AbCam at a dilution of 1:500. For f5C, we used the polyclonal antibody mix from Active Motif (61228) at a dilution of 1:500
Validation	All these commercial antibodies were quality controlled by the manufacturer using dot blot.

Eukaryotic cell lines

Policy information about cell lines

Cell line source(s)	HEK and HeLa cell lines were provided free of charge by the company Hybrigenics Services SAS
Authentication	No authentication was performed
Mycoplasma contamination	Cell lines were not tested for mycoplasma contamination
Commonly misidentified lines (See ICLAC register)	N/A
